# Strong association between higher-risk sex and HIV prevalence at the regional level: an ecological study of 27 sub-Saharan African countries

**DOI:** 10.12688/f1000research.17108.1

**Published:** 2018-12-02

**Authors:** Chris R. Kenyon, Jozefien Buyze, Ilan S. Schwartz

**Affiliations:** 1 Clinical Science, Institute of Tropical Medicine, Antwerp, 2000, Belgium; 2 University of Alberta, Alberta, Canada

**Keywords:** HIV prevalence, ecological, sexual network, high risk sex, sub-Saharan Africa

## Abstract

**Background: **It is unclear why HIV prevalence varies by nearly two orders of magnitude between regions within countries in sub-Saharan Africa. In this ecological study, we assess if HIV prevalence by region is associated with any of four markers of higher risk sexual behavior: lifetime number of partners, multiple partners in past year, higher risk sex (defined as sex with non-cohabiting, non-marital partners) and age at debut.

**Methods:** We performed Pearson’s correlation between the 4 behavioral risk factors and HIV prevalence by region in 47 nationally representative surveys from 27 sub-Saharan African countries, separately by gender. In addition, principal components analysis was used to reduce the eight risk factors (four for each gender) to two principal components (PCs). Mixed effects linear regression was used to assess the relationship between the resulting two PCs and HIV prevalence after controlling for the prevalence of male circumcision.

**Results:** HIV prevalence varied by a median 3.7 fold (IQR 2.9-7.9) between regions within countries. HIV prevalence was strongly associated with higher risk sex and, to a lesser extent, the other risk factors evaluated. Both PCs were strongly associated with HIV prevalence when assessed via linear regression.

**Conclusions:** Differences in sexual behavior may underpin the large differences in HIV-prevalence between subpopulation within sub-Saharan African countries.

## Abbreviations

DHS - Demographic Health Survey

PC - Principal components

PCA - Principal components analysis

STI - Sexually transmitted infection

## Background

*Whereas individual-level parameters may influence which individuals in a given population acquire infection, it is population-level parameters that affect the prevalence of infection*. 

                              Aral *et al*. ( Aral *et al*., 2007)

Aral and others have argued persuasively that population-level parameters such as the structure of sexual networks determine the prevalence of sexually transmitted infections (STIs) ( Aral *et al*., 2007; Morris *et al*., 2010; Morris *et al*., 2009). If this is true, then it follows that ecological studies are necessary to assess which markers of network structure are associated with higher HIV prevalence ( Kenyon & Colebunders, 2012; Morris *et al*., 2010).

Whilst a few ecological studies have found a difference in sexual behavior could explain differences in HIV prevalence ( De Walque, 2006; Kenyon *et al*., 2013; Kenyon & Colebunders, 2012; Morris *et al*., 2010; Morris *et al*., 2009), many have not ( Auvert *et al*., 2001b; Cleland & Ferry, 1995; Drain *et al*., 2004; Sawers & Stillwaggon, 2010; Wellings *et al*., 2006), and the methodologies have been questioned. For instance, most involved cross-country comparisons; these are suboptimal because national populations may be constituted by several relatively separate sexual networks ( Kenyon & Colebunders, 2013; Laumann & Youm, 1999). It would therefore be more appropriate to conduct these ecological assessments at the level of coherent sexual networks rather than national populations ( Kenyon *et al*., 2015b). If there is both a high degree of sexual partner homophily by ethnic group, and large differences in HIV prevalence between ethnic groups within countries, then it would be appropriate to use ecological analyses to assess if differences in sexual network structure could explain the variations in HIV prevalence ( Kenyon *et al*., 2013). This type of study from the USA, UK, Kenya, Uganda and South Africa has revealed positive ecological associations between various markers of sexual behavior (number of partners in past year and lifetime, partner concurrency and age of sexual debut), by ethnic group and HIV prevalence ( Kenyon *et al*., 2014a; Kenyon *et al*., 2013; Kenyon *et al*., 2014b; Kenyon *et al*., 2014c; Kenyon *et al*., 2016b; Morris *et al*., 2009).

Results from our recent ecological analysis of factors associated with HIV prevalence in Ethiopia based on data from Demographic Health Surveys (DHS) implied that it may be more appropriate to evaluate differences by region than by ethnic group ( Kenyon *et al*., 2015b). For instance, 80 different ethnic groups have been grouped into 9 ethnically-based regions in Ethiopia’s DHS surveys ( Central Statistical Agency [Ethiopia] & ICF International, 2012). Surveys from other sub-Saharan African countries also combine similar ethnic groups into regions and, as in Ethiopia, are designed to provide representative samples from each of these regions. We established that there was a high degree of sexual partner homophily by ethnic group region in Ethiopia ( Kenyon *et al*., 2015b). There was also a high (seven-fold) difference in HIV prevalence between regions and this correlated closely at an ecological level with a number of behavioral variables such as lifetime number of partners, reporting sex with a non-marital, non-cohabiting partner, and age at first sex ( Kenyon *et al*., 2015b). In this paper, we extend these earlier analyses to assess systematically if differences in HIV prevalence by region within countries in sub-Saharan Africa are associated with markers of sexual risk behavior.

## Methods

### Data source

Nationally representative HIV-serolinked DHS’s are available for 29 sub-Saharan African countries from MeasureDHS ( http://www.measuredhs.com). We limited our analyses to the 47 surveys from 27 countries where the HIV prevalence varied between regions by at least two-fold; we dropped 3/3 surveys from Lesotho, 1/1 from Swaziland and 2/3 from Zimbabwe (2005 and 2010). The selected variables were downloaded in regional format for all HIV-serolinked surveys from included countries via the STATcompiler function of MeasureDHS.

### Variables used in the analyses (number of surveys with missing data)

*Behavioral risk factors:*

*Higher risk sex (0/47)*: Percentage of women/men who have had sex with a non-marital, non-cohabiting partner in the last 12 months of all women/men reporting sexual activity in the last 12 months
*Multiple partners in past-year (0/47)*: Percentage of women/men aged 15–49 years who have had sexual intercourse with more than one partner in the last 12 months.
*Lifetime partners (11/47)*: Mean number of lifetime sexual partners among women/men who ever had sexual intercourse.
*Women/mens’ debut (5/47 for women and 30/47 for men)*: Median age at first sexual intercourse in years among women/men aged 20–49 years.

*Control variables:*

*Men circumcised (14/47)*: Percentage of men who report being circumcised.

*Outcome variable:*

*HIV prevalence (0/47):* Percentage of 15–49 year old men and women who tested positive for HIV.

These variables were selected for analysis based on associations with HIV from previous studies and coverage in a sufficient number of surveys ( Kenyon *et al*., 2014a; Kenyon *et al*., 2013; Kenyon *et al*., 2015b; Mishra *et al*., 2009; Morris *et al*., 2009).

### Statistical analysis

Our analysis used Pearson’s correlation to assess the association between lifetime partners, multiple partners in the past year, high-risk sex, sexual debut and HIV prevalence (15–49 years, men and women combined). The analyses were conducted by region within each survey and done separately for women and men. A Pearson’s *r* ≥ 0.3 or ≤ -0.3 was considered positive or negative, respectively. For each risk factor, we also compared the fold-difference in the prevalence of the risk factor between the regions with the highest and lowest HIV prevalence in each survey. 

***Multivariable analysis.*** Pearson’s correlation was used to assess the associations between the four behavioral risk factors separately between women and men. Principal components analysis (PCA) was used to reduce seven of the eight risk factors to two principal components. Sexual debut of men was not included in this process as data was missing for 30/47 surveys.

Mixed effects linear regression with a random intercept for individual surveys was then used to assess the relationship between HIV prevalence and these two principal components, controlling for prevalence of circumcision. The following sensitivity analyses were conducted. 1. Analyses stratified by gender. 2. Using principal components calculated excluding the lifetime partners of women and men variables (as these were missing in 11 surveys).

The study was conceived as being exploratory rather than hypothesis testing. Because of this and the fact that the sample sizes in each survey were relatively small, we did not use Bonferroni corrections ( Armstrong, 2014; Perneger, 1998; Rothman, 1990). A P-value below 0.05 was regarded as statistically significant. The analyses were conducted using STATA 13.0 (College Station, TX). Each DHS received ethical committee clearance for data analyses such as the one performed here. Consequently, no specific ethics committee approval was necessary for this study.

## Results

A total of 47 HIV-serolinked surveys from 27 countries were included. This sample included countries with a wide range of national HIV prevalences in 15- 49 year olds, ranging from 0.4% (95% confidence interval [CI] 0.2-0.5%) in Niger 2012 to 13.8 (95% CI, 12.9–14.8%) in Zimbabwe 2015. The HIV prevalence differed a median 3.8-fold (interquartile range [IQR] 2.9-8.5) between regions within surveys ( Table 1).

**Table 1.** Correlations between HIV prevalence and risk factors in 47 sub-Saharan African Demographic Health Surveys.

Country	Year	N regions	HIV ratio ^a^	Women				Men				
				Sexual Debut	Higher risk Sex	Lifetime partners	Multiple partners past year	Sexual Debut	Higher risk Sex	Lifetime partners	Multiple partners past year	Circumcision
**Burkina Faso**	2003	14	42	0.15	**0.61**		0.49		0.34		0.21	**-0.54**
**Burkina Faso**	2010	14	10.5	0.52	**0.86**	**0.86**	**0.75**		**0.75**	**0.60**	-0.34	-0.32
**Burundi**	2010	5	4.1		**0.95**	**0.99**	0.73		**0.99**	**0.99**	0.84	0.68
**Cameroon**	2004	12	5.2	0.44	0.56		0.52		**0.64**		0.42	**0.63**
**Cameroon**	2011	12	6	0.6	**0.72**	**0.76**	0.55		**0.63**	**0.81**	0.54	**0.70**
**Chad**	2014	20	53	**0.5**	**0.52**	0.40	0.17		**0.47**	0.37	**-0.50**	0.11
**Congo**	2009	12	3.2	0.17	-0.07	0.25	-0.09	0.14	-0.25	-0.17	-0.41	
**DRC**	2013	11	20	-0.19	-0.09	0.17	0.47	0.36	-0.03	**0.61**	0.43	
**Cote d'Ivoire**	2005	11	3.6	0.49	**0.66**	0.36	0.59	-0.27	**0.62**	0.50	**0.70**	
**Cote d'Ivoire**	2011	11	2.3	0.45	**0.61**	0.56	0.55	-0.11	**0.67**	0.47	0.49	0.03
**Ethiopia**	2005	11	30	-0.09	**0.65**		0.23		**0.87**		0.22	-0.45
**Ethiopia**	2011	11	7.2	0.14	**0.94**	**0.71**	**0.67**		**0.91**	**0.88**	0.08	-0.22
**Gabon**	2012	10	2.9	-0.44	0.38	-0.46	0.30	**0.73**	0.22	-0.52	-0.07	0.43
**Gambia**	2013	8	2.6	0.6	-0.24	-0.01	-0.09		0.18	-0.02	-0.43	
**Ghana**	2003	11	3.7	0.26	0.58		0.48		0.13		0.08	0.33
**Ghana**	2014	11	9.3	0.31	**0.97**	**0.92**	**0.67**		**0.96**	**0.91**	0.48	0.91
**Guinea**	2005	8	3	0.57	0.66		0.63		0.00		-0.36	0.68
**Guinea**	2012	8	2.7	**0.83**	0.62	0.19	0.05		**0.92**	0.33	0.02	
**Kenya**	2003	7	3.8	-0.45	0.62		**0.81**	-0.21	0.15		0.45	-0.94
**Kenya**	2008	8	15.4	**-0.84**	0.56	0.49	0.27		0.63	0.27	**0.71**	**-0.92**
**Liberia**	2007	6	4.5	-0.09	0.67	-0.13	0.49	-0.63	0.80	0.36	0.62	0.62
**Liberia**	2013	5	3.9	0.85	**0.89**	0.00	0.58	-0.26	0.72	-0.51	-0.06	0.16
**Malawi**	2004	3	2.7	-0.99	**1.00**		0.98	-0.99	0.75		-0.64	0.93
**Malawi**	2010	3	2.2	-0.64	0.97	0.99	0.99	-0.99	0.85	0.80	0.67	1.00
**Mali**	2001	7	3.6	-0.09	0.58		0.54		0.71		0.30	
**Mali**	2006	10	3.3	0.52	**0.73**	0.47	0.27		**0.71**	-0.11	0.30	0.15
**Mali**	2012	6	2.4	0.52	**0.85**	0.25	0.49		**0.83**	0.66	-0.62	-0.27
**Mozambique**	2009	11	6.8	0.47	0.48	-0.28	-0.09	0.29	0.28	0.12	0.01	-0.58
**Namibia**	2013	13	3.2	-0.18	0.13	**-0.74**	**-0.64**	-0.08	0.37	**-0.57**	0.48	-0.54
**Niger**	2006	8	5.7	0.42	0.56	0.26	-0.35		**0.82**	**0.90**	-0.23	-0.01
**Niger**	2012	8	5.5	**0.89**	**0.74**	-0.40	0.46		**0.78**	0.66	-0.44	
**Rwanda**	2005	5	3.5		**0.94**		**0.95**		**0.96**		-0.04	**0.94**
**Rwanda**	2010	5	3.5		**0.95**	**0.95**	0.80		**0.98**	**0.91**	0.69	0.86
**Rwanda**	2014	5	2.7		**0.97**	**0.95**	**0.99**		**0.97**	**0.96**	0.69	0.76
**Senegal**	2005	11	22	-0.29	**0.79**		0.37		**0.78**		0.59	-0.59
**Senegal**	2010	17	24	**-0.61**	0.22	0.20	0.39		**0.54**	0.26	**0.65**	
**Sierra Leone**	2013	14	4.9	0.4	**0.57**	0.37	-0.02	-0.13	0.35	0.02	0.08	0.46
**Tanzania**	2003	21	6.8	0.26	0.09	-0.06	-0.15		0.06	0.00	-0.15	
**Tanzania**	2007	24	52.3	-0.18	0.37	0.18	0.17		**0.46**	**0.43**	**0.43**	
**Tanzania**	2011	30	148	0.6	**0.39**	0.16	0.08		0.30	0.27	0.30	
**Togo**	2013	6	8.5	0.6	0.57	**0.90**	0.40		0.49	**0.87**	-0.05	0.18
**Uganda**	2004	9	3.7		**0.67**	0.47	0.53		0.55	0.64	0.38	
**Uganda**	2011	10	2.6	0.21	0.35	0.01	0.12	0.12	0.38	0.16	0.11	
**Zambia**	2001	9	2.7	0.51	0.21		0.40	0.03	0.44		0.41	
**Zambia**	2007	9	3.1	0.4	0.30	0.13	0.05	0.01	0.62	0.30	0.03	-0.34
**Zambia**	2013	10	2.8	0.38	0.50	0.38	**0.68**	0.12	**0.64**	0.19	0.06	0.01
**Zimbabwe**	2015	10	2	-0.23	**0.72**	**0.77**	0.47		**0.80**	**0.67**	**0.69**	0.54
**Median (IQR)**		10 (7-12)	3.8 (2.9- 8.5)	0.26 (-0.18- 0.50)	0.61 (0.38- 0.79)	0.31 (0.07- 0.73)	0.47 (0.17- 0.62)	-0.08 (-0.26- 0.12)	0.63 (0.35- 0.80)	0.40 (0.13- 0.74)	0.21 (-0.06 -0.49)	0.16 (-0.31- 0.68)

^a^HIV ratio: HIV prevalence in highest HIV prevalence region/ HIV prevalence in lowest HIV prevalence region in each survey. P<0.05 represented by bold font.

### Higher-risk sex

The prevalence of higher-risk sex between regions within surveys varied widely in women (median 4.8-fold difference [IQR 2.3-8.1]) and men (median 2.5-fold difference [IQR 1.8-5.5]).

**Figure 1.**
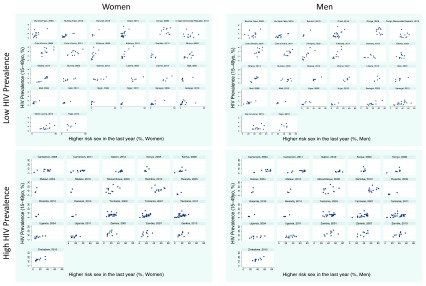
Scatterplots of HIV prevalence (15–49 year olds) versus high-risk sex behavior stratified by men/women and high/low HIV prevalence in 47 sub-Saharan African Demographic Health Surveys.

*Women*: There was a positive association between higher risk sex and HIV in 39/47 surveys, of which 26 were statistically significant. There were no surveys where the association was negative ( Table 1; Figure 1). Within surveys, the region with the highest HIV prevalence had a median 2.5 (IQR 1.4 to 6.5) times increased prevalence of higher-risk sex for women than the lowest HIV prevalence population.

*Men*: The association was positive in 38/47 surveys, 22 of which were significant, and negative in none of the surveys. In each survey, the region with the highest HIV prevalence had a median 1.9 (IQR 1.2 to 2.9) times higher prevalence of higher risk sex for men than the lowest HIV prevalence population.

### Number of lifetime sex partners

The reported number of lifetime partners between regions in each survey varied by a median 1.8-fold (IQR 1.5-2.4) for women and 2.5-fold (2.0-3.3) for men.

**Figure 2.**
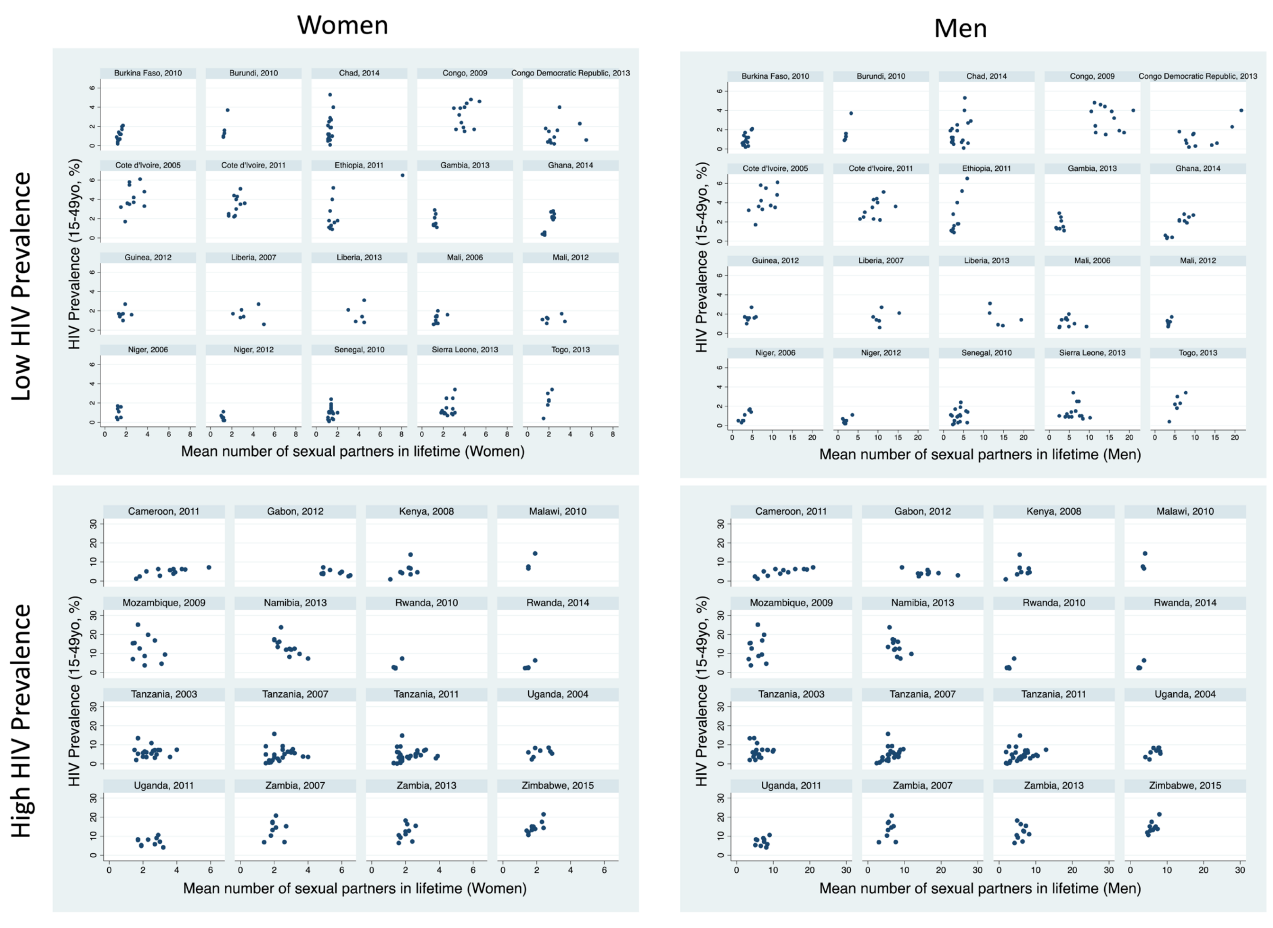
Scatterplots of HIV prevalence (15–49 year olds) versus lifetime number of sex partners stratified by men/women and high/low HIV prevalence in 47 sub Saharan African Demographic Health Surveys.

*Women*: There was a positive association between number of lifetime partners and HIV prevalence in 18/36 surveys, of which 9 were statistically significant ( Table 1, Figure 2). Of the three surveys for which the association was negative, this was statistically significant for one (Namibia, 2013). Within surveys, the region with the highest HIV prevalence had a median 1.3 (IQR 1.0 to 1.5) times higher prevalence of lifetime partners for women than the lowest HIV prevalence population.

*Men*: The association was positive in 22/36 surveys (significant in 13 surveys). In the three surveys where the association was negative, this was only significant in the case of Namibia 2013. Within each survey, the region with the highest HIV prevalence had a median 1.5 (IQR 1.1 to 2.1) times higher prevalence of lifetime partners for men than the lowest HIV prevalence population.

### Multiple partners in past-year

The percent with multiple partners in the past year varied between regions by a median 6.5-fold (IQR 3.5-13.7) for women and 2.8-fold (IQR 2.0-4.8) for men.

**Figure 3.**
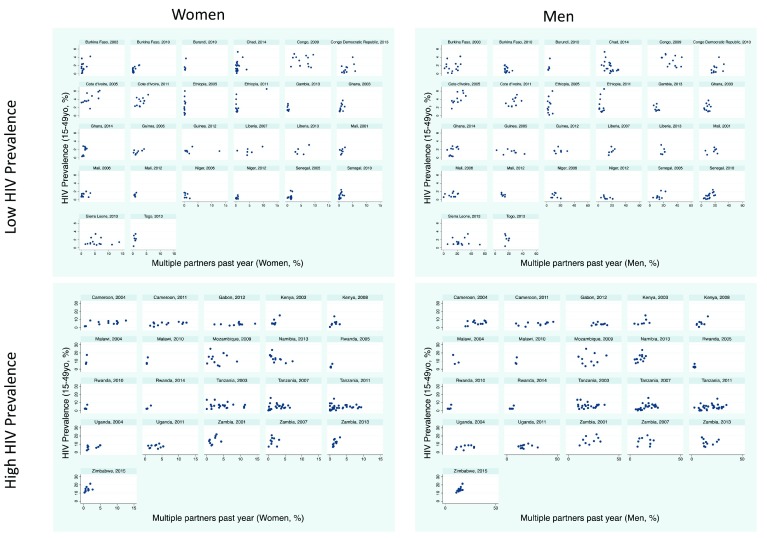
Scatterplots of HIV prevalence (15–49 year olds) versus multiple partners past year stratified by men/women and high/low HIV prevalence in 47 sub Saharan African Demographic Health Surveys.

*Women*: There was evidence of a positive association in 30/47 surveys but this association was only significant in five surveys ( Table 1, Figure 3). The association was negative in three surveys, one of which was significant (Namibia, 2013). In each survey, the region with the highest HIV prevalence had a median 2.1 (IQR 1.4 to 4.1) times higher prevalence of multiple partners for women than the lowest HIV prevalence population.

*Men*: The association was positive in 21/47 surveys, of which four were statistically significant and negative in 10 surveys, of which one was significant – Chad 2014. Within surveys, the region with the highest HIV prevalence had a median 1.3 (IQR 1.0 to 2.1) times higher prevalence of multiple partner for men than the lowest HIV prevalence population.

### Men’s and women’s debut

**Figure 4.**
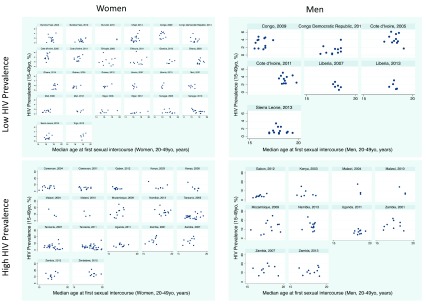
Scatterplots of HIV prevalence (15–49 year olds) versus median age of sexual debut stratified by men/women and high/low HIV prevalence in 47 sub Saharan African Demographic Health Surveys.

*Women*: There was evidence of a positive association between women’s age at sexual debut and HIV prevalence in 22/42 surveys, but this association was only significant in three surveys (Chad, 2014; Guinea, 2012; Niger, 2012; Table 1, Figure 4). The association was negative in six surveys, of which this was significant in two (Kenya, 2008; Senegal, 2010).

*Men*: The association was positive in 1/17 surveys, which was statistically significant (Gabon, 2012) and negative in three surveys, none of which was significant.

### Associations between behavioral risk factors

Within both women and men, the three behavioral risk factors (lifetime partners, past year partners and high-risk sex) were positively associated with one another ( Table 2). Within men (but not women) these three risk factors were negatively associated with age at first sex.

**Table 2.** Correlations between behavioral risk factors in 47 sub-Saharan African Demographic Health Surveys.

				Women										Men										
				Correlations with HIV Prevalence				Correlations between behavioral factors						Correlations with HIV Prevalence					Correlations between behavioral factors					
Country	Year	N regions	HIV ratio	Debut	Higher risk Sex	Lifetime partners	Multiple partners past year	Lifetime partners vs Higher risk sex	Multiple partners past year vs Higher risk sex	Multiple partners past year vs lifetime partners	Debut vs Higher risk Sex	Debut vs Lifetime partners	Debut vs Multiple partners past year	Debut	Higher risk Sex	Lifetime partners	Multiple partners past year	Circumcision	Lifetime partners vs Higher risk sex	Multiple partners past year vs Higher risk sex	Multiple partners past year vs lifetime partners	Debut vs Higher risk Sex	Debut vs Lifetime partners	Debut vs Multiple partners past year
**Burkina Faso**	2003	14	42	0.15	**0.61**		0.49		**0.9**		**0.55**		0.51		0.34		0.21	**-0.54**		**0.63**				
**Burkina Faso**	2010	14	10.5	0.52	**0.86**	**0.86**	**0.75**	**0.81**	**0.89**	**0.81**	**0.67**	0.41	**0.59**		**0.75**	**0.60**	-0.34	-0.32	**0.78**	-0.14	-0.10			
**Burundi**	2010	5	4.1		**0.95**	**0.99**	0.73	**0.96**	**0.9**	0.78					**0.99**	**0.99**	0.84	0.68	**0.98**	0.79	0.86			
**Cameroon**	2004	12	5.2	0.44	0.56		0.52		**0.8**		**0.83**		0.43		**0.64**		0.42	**0.63**		**0.78**				
**Cameroon**	2011	12	6	0.6	**0.72**	**0.76**	0.55	**0.81**	**0.8**	**0.83**	**0.62**	0.17	0.46		**0.63**	**0.81**	0.54	**0.70**	**0.77**	**0.82**	**0.88**			
**Chad**	2014	20	53	**0.5**	**0.52**	0.40	0.17	**0.8**	**0.57**	**0.72**	**0.59**	0.33	0.23		**0.47**	0.37	**-0.50**	0.11	**0.77**	-0.44	-0.10			
**Congo**	2009	12	3.2	0.17	-0.07	0.25	-0.09	0.72	**0.78**	0.56	0.38	0.01	0.23	0.14	-0.25	-0.17	-0.41		0.57	**0.75**	**0.77**	-0.18	-0.51	-0.37
**DRC**	2013	11	20	-0.19	-0.09	0.17	0.47	0.35	0.21	**0.86**	0.21	**-0.68**	-0.59	0.36	-0.03	**0.61**	0.43		0.22	0.43	**0.80**	-0.51	-0.30	-0.42
**Cote d'Ivoire**	2005	11	3.6	0.49	**0.66**	0.36	0.59	**0.63**	0.52	0.29	0.46	-0.11	0.34	-0.27	**0.62**	0.50	**0.70**		**0.70**	**0.73**	**0.70**	-0.23	**-0.77**	-0.41
**Cote d'Ivoire**	2011	11	2.3	0.45	**0.61**	0.56	0.55	0.6	0.54	**0.63**	0.46	0.03	0.03	-0.11	**0.67**	0.47	0.49	0.03	**0.79**	0.53	**0.63**	-0.40	-0.47	-0.07
**Ethiopia**	2005	11	30	-0.09	**0.65**		0.23		0.17		-0.05		0.08		**0.87**		0.22	-0.45		0.11				
**Ethiopia**	2011	11	7.2	0.14	**0.94**	**0.71**	**0.67**	**0.85**	**0.82**	**1.00**	-0.06	-0.20	-0.18		**0.91**	**0.88**	0.08	-0.22	**0.92**	0.10	0.25			
**Gabon**	2012	10	2.9	-0.44	0.38	-0.46	0.30	-0.01	**0.84**	-0.04	0.15	-0.31	0.29	**0.73**	0.22	-0.52	-0.07	0.43	0.60	0.47	0.55	0.13	-0.50	-0.17
**Gambia**	2013	8	2.6	0.6	-0.24	-0.01	-0.09	**0.86**	**0.94**	**0.72**	0.78	**0.91**	0.47		0.18	-0.02	-0.43		**0.96**	-0.76	**-0.76**			
**Ghana**	2003	11	3.7	0.26	0.58		0.48		0.57		0.07		0.26		0.13		0.08	0.33		**0.82**				
**Ghana**	2014	11	9.3	0.31	**0.97**	**0.92**	**0.67**	**0.9**	**0.79**	**0.71**	0.33	0.23	0.31		**0.96**	**0.91**	0.48	0.91	**0.95**	**0.63**	0.49			
**Guinea**	2005	8	3	0.57	0.66		0.63		**0.97**		**0.81**		**0.81**		0.00		-0.36	0.68		0.41				
**Guinea**	2012	8	2.7	**0.83**	0.62	0.19	0.05	0.78	0.58	**0.93**	0.44	-0.06	-0.20		**0.92**	0.33	0.02		0.42	0.10	**0.74**			
**Kenya**	2003	7	3.8	-0.45	0.62		**0.81**		0.4		0.23		-0.72	-0.21	0.15		0.45	-0.94		**0.89**		-0.35		-0.17
**Kenya**	2008	8	15.4	**-0.84**	0.56	0.49	0.27	0.48	0.55	0.42	-0.36	-0.29	-0.20		0.63	0.27	**0.71**	**-0.92**	**0.79**	0.16	0.14			
**Liberia**	2007	6	4.5	-0.09	0.67	-0.13	0.49	0.31	0.59	0.58	0.19	-0.03	0.42	-0.63	0.80	0.36	0.62	0.62	0.10	0.65	0.52	-0.76	-0.60	-0.78
**Liberia**	2013	5	3.9	0.85	**0.89**	0.00	0.58	0.21	0.87	0.53	**0.98**	0.12	0.87	-0.26	0.72	-0.51	-0.06	0.16	-0.18	0.60	0.52	-0.68	-0.59	-0.82
**Malawi**	2004	3	2.7	-0.99	**1.00**		0.98		0.99		**-1.00**		-0.99	-0.99	0.75		-0.64	0.93		0.03		-0.65		0.74
**Malawi**	2010	3	2.2	-0.64	0.97	0.99	0.99	0.99	0.99	**1**	-0.82	-0.73	-0.73	-0.99	0.85	0.80	0.67	1.00	1.00	0.96	0.98	-0.91	-0.87	-0.76
**Mali**	2001	7	3.6	-0.09	0.58		0.54		0.76		0.60		0.59		0.71		0.30			0.55				
**Mali**	2006	10	3.3	0.52	**0.73**	0.47	0.27	0.05	0.24	0.58	0.55	0.22	-0.19		**0.71**	-0.11	0.30	0.15	0.32	0.53	0.25			
**Mali**	2012	6	2.4	0.52	**0.85**	0.25	0.49	0.62	0.82	0.63	0.68	0.26	**0.88**		**0.83**	0.66	-0.62	-0.27	**0.84**	-0.56	-0.07			
**Mozambique**	2009	11	6.8	0.47	0.48	-0.28	-0.09	0.45	0.59	**0.86**	0.36	-0.48	-0.34	0.29	0.28	0.12	0.01	-0.58	**0.77**	**0.91**	**0.80**	-0.50	-0.60	-0.48
**Namibia**	2013	13	3.2	-0.18	0.13	**-0.74**	**-0.64**	-0.35	-0.16	**0.82**	**0.74**	-0.11	-0.14	-0.08	0.37	**-0.57**	0.48	-0.54	-0.31	**0.77**	-0.11	0.28	-0.54	-0.21
**Niger**	2006	8	5.7	0.42	0.56	0.26	-0.35	0.06	0.21	-0.25	**0.93**	-0.14	0.24		**0.82**	**0.90**	-0.23	-0.01	**0.90**	-0.19	-0.06			
**Niger**	2012	8	5.5	**0.89**	**0.74**	-0.40	0.46	-0.29	0.24	-0.46	**0.93**	-0.28	0.34		**0.78**	0.66	-0.44		**0.85**	-0.47	-0.05			
**Rwanda**	2005	5	3.5		**0.94**		**0.95**		**0.94**						**0.96**		-0.04	**0.94**		0.03				
**Rwanda**	2010	5	3.5		**0.95**	**0.95**	0.80	**0.99**	0.8	0.79					**0.98**	**0.91**	0.69	0.86	**0.97**	0.75	0.84			
**Rwanda**	2014	5	2.7		**0.97**	**0.95**	**0.99**	**0.97**	**0.99**	**0.98**					**0.97**	**0.96**	0.69	0.76	**0.99**	0.77	0.83			
**Senegal**	2005	11	22	-0.29	**0.79**		0.37		0.32		-0.08		-0.24		**0.78**		0.59	-0.59		**0.62**				
**Senegal**	2010	17	24	**-0.61**	0.22	0.20	0.39	**0.78**	**0.89**	**0.78**	-0.01	0.04	-0.41		**0.54**	0.26	**0.65**		**0.67**	**0.68**	0.21			
**Sierra Leone**	2013	14	4.9	0.4	**0.57**	0.37	-0.02	0.48	0.45	**0.70**	**0.82**	0.46	0.47	-0.13	0.35	0.02	0.08	0.46	**0.55**	**0.85**	**0.63**	-0.51	**-0.81**	**-0.65**
**Tanzania**	2003	21	6.8	0.26	0.09	-0.06	-0.15	**0.71**	**0.71**	**0.79**	**-0.50**	**-0.77**	**-0.75**		0.06	0.00	-0.15		**0.69**	**0.76**	**0.75**			
**Tanzania**	2007	24	52.3	-0.18	0.37	0.18	0.17	**0.76**	**0.78**	**0.83**	-0.39	**-0.68**	**-0.72**		**0.46**	**0.43**	**0.43**		**0.79**	**0.71**	**0.77**			
**Tanzania**	2011	30	148	0.6	**0.39**	0.16	0.08	**0.83**	**0.77**	**0.86**	**-0.53**	**-0.65**	**-0.74**		0.30	0.27	0.30		**0.81**	**0.80**	**0.68**			
**Togo**	2013	6	8.5	0.6	0.57	**0.90**	0.40	**0.82**	**0.82**	0.68	0.74	0.60	0.42		0.49	**0.87**	-0.05	0.18	0.56	0.35	0.11			
**Uganda**	2004	9	3.7		**0.67**	0.47	0.53	**0.76**	**0.9**	**0.91**					0.55	0.64	0.38		**0.81**	**0.78**	**0.88**			
**Uganda**	2011	10	2.6	0.21	0.35	0.01	0.12	**0.75**	**0.88**	**0.89**	-0.21	-0.58	-0.55	0.12	0.38	0.16	0.11		**0.72**	0.36	0.55	-0.54	**-0.67**	**-0.74**
**Zambia**	2001	9	2.7	0.51	0.21		0.40		**0.87**		-0.47		-0.04	0.03	0.44		0.41			**0.71**		-0.61		-0.18
**Zambia**	2007	9	3.1	0.4	0.30	0.13	0.05	**0.88**	**0.82**	**0.90**	-0.51	**-0.79**	**-0.74**	0.01	0.62	0.30	0.03	-0.34	**0.73**	0.65	**0.68**	-0.40	**-0.78**	-0.66
**Zambia**	2013	10	2.8	0.38	0.50	0.38	**0.68**	**0.92**	0.11	0.17	-0.41	-0.37	0.46	0.12	**0.64**	0.19	0.06	0.01	0.44	0.54	0.22	-0.49	-0.62	-0.57
**Zimbabwe**	2015	10	2	-0.23	**0.72**	**0.77**	0.47	**0.94**	**0.87**	**0.78**	0.31	0.10	0.46		**0.80**	**0.67**	**0.69**	0.54	**0.76**	0.60	**0.83**			
**Median (IQR)**		10 (7- 12)	3.8 (2.9- 8.5)	0.26(- 0.18- 0.50)	0.61(0.38- 0.79)	0.31 (0.07- 0.73)	0.47(0.17- 0.62)	0.76 (0.54- 0.88)	0.79 (0.54- 0.88)	0.78 (0.58- 0.85)	0.34 (-0.08- 0.67)	-0.08 ('-0.42- 0.19)	0.22 ('-0.34- 0.46)	-0.08 (-0.26- 0.12)	0.63(0.35- 0.80)	0.40(0.13- 0.74)	0.21(- 0.06- 0.49)	0.16(-0.31- 0.68)	0.77 (0.57- 0.84)	0.61 (0.15- 0.77)	0.59 (0.18- 0.79)	-0.50 (-0.61-- 035)	-0.60 (-0.77-- 0.51)	-0.42 (-0.66- -0.18)

### Multivariable analyses

**Figure 5.**
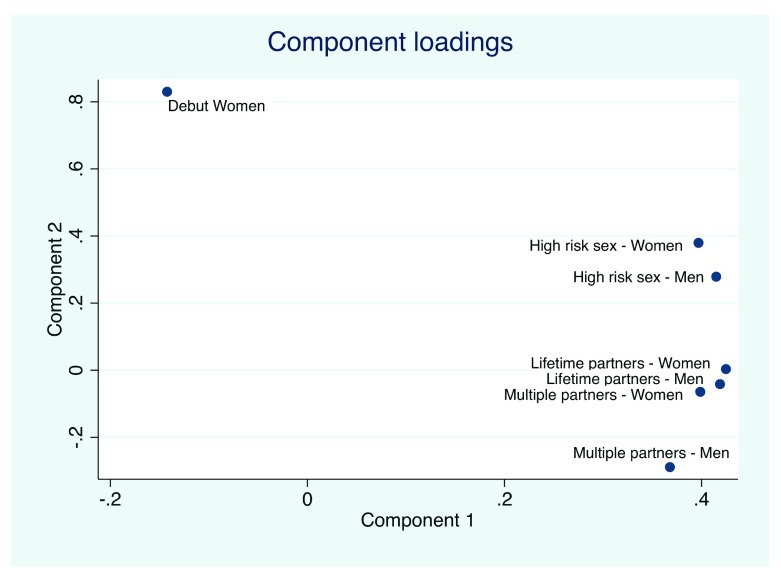
Loadings of the 7 behavioral risk factors in principal components 1 and 2.

*Principal components*: Two principal components were able to explain 80% of the variation in the seven variables. The component loadings of the principal components are depicted in Figure 5. Principal component one (PC1) represented a summation of the variables high-risk sex (women and men), lifetime partners (women and men) and multiple partners (women and men), minus the variable debut age women ( Figure 5).

In the unadjusted model, PC1 and PC2 were associated with HIV prevalence (coeff. 0.51, 95% CI 0.31-0.71, and coeff. 0.72, 95% CI 0.38-1.05, both P<0.005, respectively; Table 3). Adjusting for the prevalence of circumcision made little difference to the strength of the association ( Table 3).

**Table 3.** Mixed effects multiple regression with HIV-prevalence by region as outcome variable [Coefficient (95% CI)].

PC	Including lifetime sex partners		Excluding lifetime sex partners	
	Unadj.	Adj. Circumcision	Unadj.	Adj. Circumcision
**PC1**	0.51 (0.31-0.71) **	0.76 (.49-1.03) **	0.64 (.44-.84) **	0.96 (.71-1.21) **
**PC2**	0.72 (0.38-1.05) **	0.58 (0.14-1.0) **	0.55 (.26-.84) **	0.39 (.04-.73) *
**Circumcision**	-	-0.09 (-.11--.07) **	-	-0.09 (-.11--.07) **

*P<0.05, **P<0.005. PC, principal component.

Sensitivity analyses repeating the analyses stratified by gender, using the principal components that were calculated excluding the lifetime partners (women and men) variables in the analyses made little difference to the results ( Table 3).

## Discussion

We found wide variations in the prevalence of HIV and the four behavioral risk factors evaluated. All the risk factors, excluding women’s/men’s debut, were positively associated with one another and with HIV prevalence. Combining the four risk factors via principal components analysis generated two components which were strongly associated with HIV prevalence.

There are a number of interpretations of and limitations to our results. Demographic and Health Surveys are not designed to accurately assess sensitive information such as sexual behavior ( Beauclair *et al*., 2013; Morris *et al*., 2014). As such, they have been shown to underestimate the prevalence of behaviors that may be particularly sensitive to a respondent bias, such as number of partners ( Glick & Sahn, 2007; Kenyon *et al*., 2013; Morris *et al*., 2014). There is, however, no evidence that we could find that this bias would operate differentially by ethnic or regional group ( Johnson *et al*., 2009; Kenyon *et al*., 2014a). This bias should thus result in underestimated the prevalence of reported risk behavior, but this effect should not differ by region. We did not control for a broad range of variables that may confound our results. Age of respondents, which is known to affect HIV prevalence and sexual behaviors such as lifetime partner number, was not controlled for. However, the DHS sampling strategy typically produces populations that do not differ by age between regions ( Central Statistical Agency [Ethiopia] & ICF International, 2012; De Walque, 2006; Kenyon *et al*., 2013; Kenyon *et al*., 2015b). We also decided not to control for condom usage or upstream determinants such as socioeconomic status. In keeping with a number of other African studies ( Kenyon *et al*., 2014a; Kenyon *et al*., 2013; Kenyon *et al*., 2015b), we found that condom usage tended to be higher in areas more affected by HIV (data not shown). As a result, we did not control for condom usage. 

**Figure 6.**
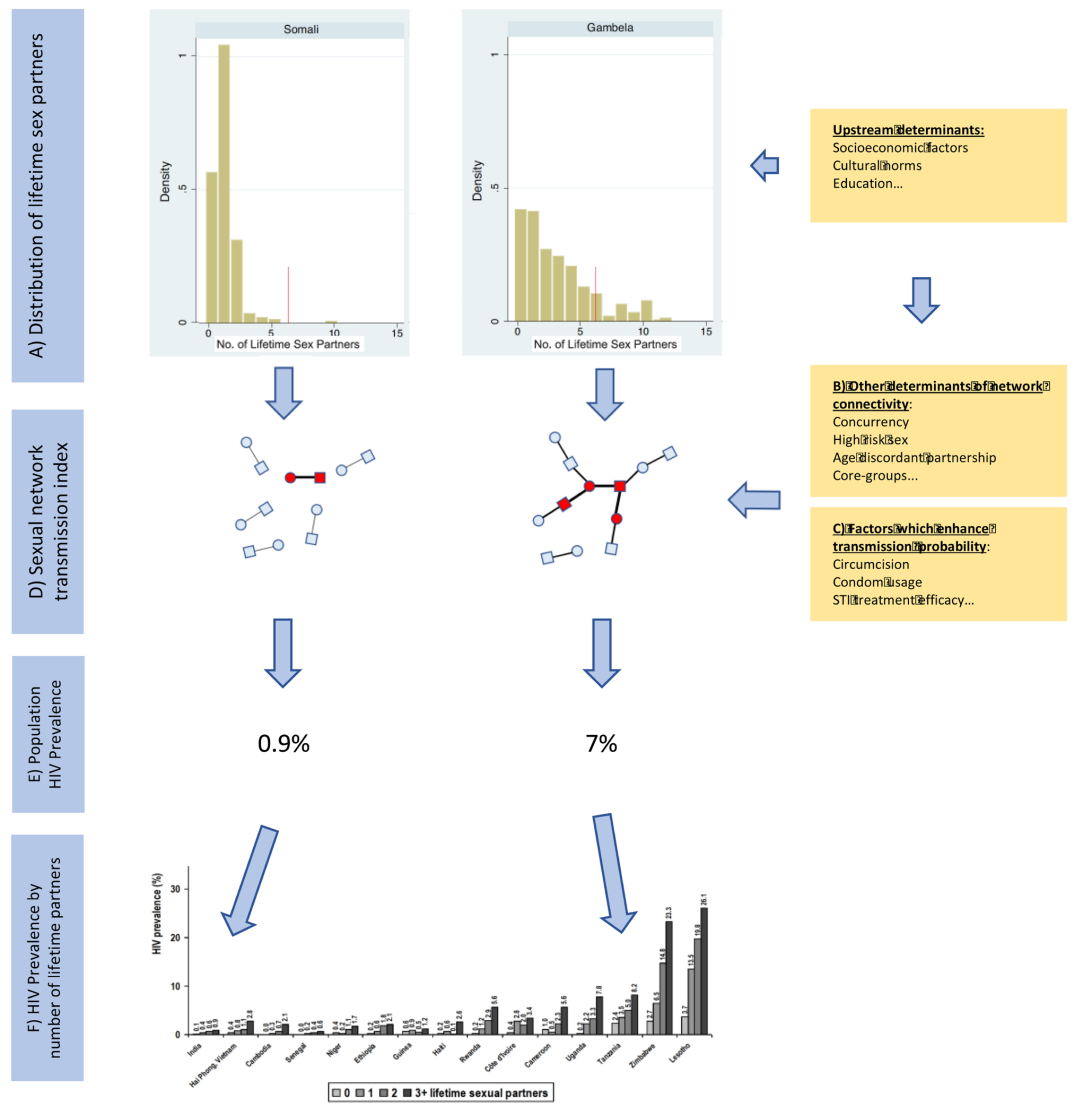
Conceptual framework for understanding how risk factors could come together to produce sexual networks that are higher risk for HIV transmission in certain populations than others, using the example of Ethiopian regions. ( **A**) Men in Gambela (HIV prevalence 7%) report a higher number of lifetime sex partners (both median and percent with more than 6 partners – vertical red line) than those in the Somali region (HIV prevalence 0.9%) (Reproduced from [14] with permission). ( **B**) This in conjunction with other determinants of enhanced network connectivity will result in a larger reachable path for HIV in Gambela than Somali. The reachable path is represented by the edges between nodes in the sexual networks. ( **C**) The prevalence of other risk factors such as condom usage and circumcision influence the probability of HIV transmission per sexual contact (probability of transmission is depicted as proportional to edge width between nodes). ( **D**) The composite of A, B and C determine the sexual network transmission index, which combines the reachable path determined by A and B with the probability of transmission ( **C**) and results in a higher HIV prevalence (red nodes) in Gambela than Somali ( **E**). Within both Gambela and Somali there is a stepwise increase in HIV prevalence with increasing number of partners, but given the higher sexual network transmission index in Gambela, the probability of HIV is higher for someone with a low number of partners in Gambela than someone with a high number of partners in Somali. ( **F**) represents the HIV prevalence by number of lifetime sex partners for men 15–49 years from all DHS countries with available data. Graphic reproduced from [20] with permission. Somali most closely approximates India and Gambela, Tanzania.

The relationship between socioeconomic status and HIV is complex, with most evidence pointing to a positive association between wealth and HIV in sub-Saharan Africa ( Hajizadeh *et al*., 2014; Mishra *et al*., 2009; Susser, 1994). We found the same to be true at regional level (data not shown). Because our conceptual framework ( Figure 6) conceived the upstream determinants as operating via sexual behavior, we considered it would be inappropriate to control for these in our model. The surveys were done at different stages in the HIV epidemics of the various countries. A wide range of studies have found that populations in sub-Saharan Africa have responded to the HIV epidemic by reducing a range of risk behaviors including casual partners and partner numbers ( Glick & Sahn, 2007; Hajizadeh *et al*., 2014; Halperin *et al*., 2011; Kirby, 2008). By preferentially affecting persons with higher numbers of partners, AIDS mortality may also reduce the average number of partners reported in high prevalence settings ( Chesson *et al.*, 2003; Kenyon *et al*., 2016a). Not controlling for age of the epidemic, behavior change and AIDS mortalities, effect on partner number should, however, serve to dilute any relationship between sexual risk behaviors and HIV prevalence. We did not assess heterogeneity in HIV prevalence and high-risk behaviors within regions or the extent to which sexual networks were coterminous with regions within countries. Once again unconsidered heterogeneity would be expected to reduce the strength of the association between HIV and risk-behavior.

Because HIV prevalence is a product of a number of risk factors operating over decades, one would not expect to consistently find a linear relationship between HIV prevalence and any particular risk factor by region. For this reason, in additional to the Pearson’s correlations, we compared the prevalence of each of the risk factors between the lowest and highest HIV prevalence region in each survey. These provided results commensurate with those provided by linear regression. If non-participation in the surveys or the HIV-testing component were not random this could result in non-response biases. However, response rates in most of the surveys were high. This study involves the ecological analysis of cross sectional data. As such we can only describe associations found and not attribute causation.

Convincing evidence of a strong link between rate of partner change and risk of STIs including HIV has been provided by empirical individual level studies, modelling studies, and the theoretical importance of rate of partner change in the formula for the basic reproductive number ( Auvert *et al*., 2001a; Chen *et al*., 2007; Mishra *et al*., 2009; Xu *et al*., 2006). Women reporting sex with a non-marital, non-cohabiting person have also been shown to be more likely to be HIV positive in all of 18 DHS’s where this was assessed ( Mishra *et al*., 2009). The same was true for men in only 1/18 surveys (Vietnam) ( Mishra *et al*., 2009). These findings at an individual level reduce the chance that the association we found between these same factors and HIV at an ecological level is due to an ecological inference fallacy. Furthermore, studies from nationally representative samples in several sub-Saharan African countries have found that region and/or ethnic group remain strongly associated with HIV after controlling for a range of standard individual level risk factors (adjusted odds ratios of up to 14.3 (95% CI 6.1- 33.4)( Barankanira *et al*., 2016; Fraser-Hurt *et al*., 2011; Johnson & Way, 2006; Johnson & Budlender, 2002; Mermin *et al*., 2008; Oluoch *et al*., 2011). This suggests that there is a risk factor at the level of region and/or ethnic group that was not adequately controlled for in these analyses. This risk factor could be a wide range of factors, such as HSV-2 prevalence ( Weiss *et al*., 2001), composition of the vaginal microbiome ( Buve *et al*., 2014), host genetic factors ( Ramsuran *et al*., 2011) or differential network connectivity ( Halperin *et al*., 2011; Kenyon *et al*., 2013; Kirby, 2008). Our ecological study is unable to assess which of these factors is responsible for the variations in HIV-prevalence by region. However, our results are compatible with the network-connectivity theory ( Kenyon *et al.*, 2017). In brief, this theory posits that populations vary in how connected their sexual networks are (determined by factors such as rate of partner change, concurrency). This, combined with the prevalence of other risk factors that affect the probability of transmission per contact (such as condom use, circumcision and STI prevalence), determines the prevalence of HIV ( Kenyon *et al*., 2017) (see Figure 6 for a more detailed explanation). Why was higher risk sex the behavioral risk factor most strongly associated with HIV-prevalence? Having sex with a non-marital, non-cohabiting person may be an independent risk factor but it may also be associated with another risk behavior that was either unmeasured or inaccurately measured and this other risk behavior is a driver of HIV transmission. Given that the DHS methodology is poor at ascertaining socially sensitive information such as respondent concurrency, questions regarding less sensitive information (such as if a partner was non-marital, non-cohabiting) may be more accurately ascertained. 

## Conclusion

The available evidence, including the results from this study, suggests that a variety of combinations of behavioural and other risk factors result in high HIV prevalence ( Buve, 2006; Kenyon *et al*., 2014a; Kenyon *et al*., 2013; Kenyon *et al*., 2015b; Laumann & Youm, 1999). A striking finding of this study was the strong positive associations between lifetime partners, multiple partners and higher risk sex and the negative association with debut in men. These associations suggest that these risk factors may be underpinned by a common factor. Evidence of varying strengths has been advanced for a wide range of upstream determinants including demographic, socioeconomic and norm-related factors ( Abramsky *et al*., 2014; Barnighausen *et al.*, 2007; Bowleg *et al*., 2011; Carter *et al*., 2007; Chen *et al*., 2007; Gorbach *et al.*, 2002; Kenyon *et al*., 2014a; Kenyon *et al*., 2015a; Leclerc-Madlala, 2009; Mulawa *et al*., 2016; Richardson *et al*., 2014; Yamanis *et al*., 2016). More research is required to better delineate the relationship between these upstream factors, sexual-behaviors and the resulting sexual-networks and HIV prevalence. Future behavioral and HIV surveys would be strengthened by using audio-computer-assisted self-interview technology to collect sexual behavioral information ( Le & Vu, 2012). In countries with large variations in HIV–prevalence thought could be given to using the sexual behaviour of the lower HIV-prevalence communities as positive examples for what could be achieved with behavior change in high HIV-prevalence communities ( The Positive Deviance Approach in Female Genital Mutilation Eradication, 1999). 

## Data availability

The datasets analyzed during the current study are available in the MEASURE DHS repository, ( http://www.measuredhs.com). Access to the dataset requires registration, and is granted to those that wish to use the data for legitimate research purposes. A guide for how to apply for dataset access is available at: https://dhsprogram.com/data/Access-Instructions.cfm. The exact datasets analyzed are detailed in Table 1.
