# Supplementary material for: Strong association between higher-risk sex and HIV prevalence at the regional level: an ecological study of 27 sub-Saharan African countries
Source: F1000Res. 2018 Dec 2;7:1879. [Version 1] doi: 10.12688/f1000research.17108.1 (PMC6367661; doi:10.12688/f1000research.17108.1)
Supplement: Peer review history

# Peer review: Referee response for version 1

**DOI:** 10.5256/f1000research.18706.r43673

**Published:** 2019-02-06

**Authors:** Brian G. Williams

**Affiliations:** 1 South African Centre for Epidemiological Modelling and Analysis (SACEMA), Stellenbosch University, Stellenbosch, South Africa

The authors carry out a detailed analysis of the association between risky sex and HIV prevalence at the regional level. They show that the prevalence of HIV is associated with risky sex. While this result is not unexpected it is important to quantify the relationship and to examine how the prevalence of HIV and risky sex vary among regions within countries.

 However, in my view a critical factor in determining the timing and peak prevalence of HIV epidemics depends critically on how people move. It is trivial to observe that sexually transmitted infections depend on direct, intimate contact between two individuals. If people don't move then, by definition, the epidemic cannot spread. 

 DHS studies do not, of course, capture information on migration, and in particular oscillating migration, as is common in South Africa and its neighbouring countries and territories, and a direct consequence of the needs of the mining industry for a cheap supply of very large numbers of unskilled men rooted in the system of Apartheid. I would recommend reading this paper in parallel with the paper by John Hargrove "Migration, mines and mores: the HIV epidemic in southern Africa" published in the South African Journal of Science ^1^.

I have read this submission. I believe that I have an appropriate level of expertise to confirm that it is of an acceptable scientific standard.

## References

[1] Hargrove, J: Migration, mines and mores: the HIV epidemic in southern Africa. *S. Afr. j. sci.*.2008;104:53-61

# Peer review: Referee response for version 1

**DOI:** 10.5256/f1000research.18706.r42625

**Published:** 2019-01-09

**Authors:** Nico Nagelkerke

**Affiliations:** 1 Department of Medical Microbiology, University of Manitoba, Winnipeg, Canada

Kenyon and his coauthors explore whether self reported sexual "risk" taking on an aggregate level is associated with HIV prevalence. They present cogent arguments, supported by an extensive theoretical literature on sexual networks and propagation of STIs along such networks, why this is a valuable approach. I agree, but before reading their paper would have been pessimistic about the outcome because of the well-known misreporting of sexual behavior. That they nonetheless find some moderate to strong associations between (aggregated) self-reported risk behavior and HIV prevalence is not just a (methodological) justification of their approach but also (finally perhaps) a rationale for asking such behavior questions (in DHS and other surveys). 

 The paper is well written with appropriate figures and tables. All potential weaknesses were - as far as I could tell - recognized, acknowledged, and addressed by the authors. As regards to the statistical methods: although alternative approaches were possible, their approach was not flawed, and the choice of methods seemed appropriate.

I have read this submission. I believe that I have an appropriate level of expertise to confirm that it is of an acceptable scientific standard.
